# Basic mechanism of the autonomous ClpG disaggregase

**DOI:** 10.1016/j.jbc.2021.100460

**Published:** 2021-02-24

**Authors:** Panagiotis Katikaridis, Ute Römling, Axel Mogk

**Affiliations:** 1 Center for Molecular Biology of Heidelberg University (ZMBH), DKFZ-ZMBH Alliance, Heidelberg, Germany; 2 German Cancer Research Center (DKFZ), A250 Chaperones and Proteases, Heidelberg, Germany; 3 Department of Microbiology, Tumor and Cell Biology, Karolinska Institute, Stockholm, Sweden

**Keywords:** ATPase associated with diverse cellular activities (AAA+), protein aggregation, molecular chaperone, stress, 70-kDa heat shock protein (Hsp70), AAA+, ATPase associated with diverse cellular activities, CTE, C-terminal extension, Hsp70, 70-kDa heat shock protein, Hsp100, 100-kDa heat shock protein, M-domain, middle domain, MDH, malate dehydrogenase, N-domain, N-terminal domain, PVDF, polyvinylidene difluoride, SEC, size-exclusion chromatography, TBS, Tris-buffered saline

## Abstract

Bacterial survival during lethal heat stress relies on the cellular ability to reactivate aggregated proteins. This activity is typically executed by the canonical 70-kDa heat shock protein (Hsp70)–ClpB bichaperone disaggregase, which is most widespread in bacteria. The ClpB disaggregase is a member of the ATPase associated with diverse cellular activities protein family and exhibits an ATP-driven threading activity. Substrate binding and stimulation of ATP hydrolysis depends on the Hsp70 partner, which initiates the disaggregation reaction. Recently elevated heat resistance in gamma-proteobacterial species was shown to be mediated by the ATPase associated with diverse cellular activities protein ClpG as an alternative disaggregase. *Pseudomonas aeruginosa* ClpG functions autonomously and does not cooperate with Hsp70 for substrate binding, enhanced ATPase activity, and disaggregation. With the underlying molecular basis largely unknown, the fundamental differences in ClpG- and ClpB-dependent disaggregation are reflected by the presence of sequence alterations and additional ClpG-specific domains. By analyzing the effects of mutants lacking ClpG-specific domains and harboring mutations in conserved motifs implicated in ATP hydrolysis and substrate threading, we show that the N-terminal, ClpG-specific N1 domain generally mediates protein aggregate binding as the molecular basis of autonomous disaggregation activity. Peptide substrate binding strongly stimulates ClpG ATPase activity by overriding repression by the N-terminal N1 and N2 domains. High ATPase activity requires two functional nucleotide binding domains and drives substrate threading which ultimately extracts polypeptides from the aggregate. ClpG ATPase and disaggregation activity is thereby directly controlled by substrate availability.

Bacterial 100-kDa heat shock protein (Hsp100) chaperones constitute a subfamily of ATPase associated with diverse cellular activities (AAA+) proteins and play crucial roles in proteostasis networks by acting on misfolded and aggregated proteins (1). They consist of one or two AAA domains, which mediate ATP binding and hydrolysis and oligomerization into hexameric rings. The energy derived from ATP hydrolysis is used to thread substrate proteins through an inner translocation channel. Substrate-binding pore residues of the AAA domains are arranged in a spiral staircase and propel the substrate in discrete steps that are orchestrated by cycles of sequential ATP hydrolysis events (2). Although AAA+ proteins share this basic motor activity, they differ substantially in cellular functions and act on diverse substrates. Functional specificity is gained by extra domains, which are either fused to or are inserted into the AAA module (3, 4). These extra domains either directly interact with specific substrates or act as binding platforms for cooperating adaptor proteins, which deliver their bound cargo for processing by their AAA+ partner proteins (5). Extra domains can additionally regulate ATPase activity of AAA+ proteins, allowing to adjust AAA+ motor activity to partner and substrate availability (6).

Hsp100 disaggregases solubilize aggregated proteins and protect cells during severe heat stress, which causes protein misfolding and aggregation (6). Hsp100 disaggregases are composed of two AAA domains (AAA1 and AAA2) but differ with respect to composition of extra domains. The most widespread bacterial disaggregation system is composed of the AAA+ protein ClpB, which cooperates with a cognate 70-kDa heat shock protein (Hsp70) (DnaK) chaperone system (7, 8, 9). ClpB harbors two extra domains, an N-terminal domain (N domain) and a coiled-coil middle domain (M domain) that is inserted into the AAA1 module (10). While the N domain fulfills an auxiliary function, the M domain is essential for disaggregation as it mediates the direct interaction and cooperation with the Hsp70 partner (11, 12, 13). This allows recruitment of ClpB to the aggregate surface, as ClpB does not directly bind to protein aggregates but requires Hsp70 for targeting (14, 15). M domains additionally tightly regulate the ClpB AAA+ motor, mediating its repression in aggregate-loaded Hsp70 and allowing for its activation upon disaggregase recruitment to protein aggregates (16, 17, 18).

ClpG (ClpK) constitutes an alternative disaggregase, which confers enhanced stress resistance including lethal heat resistance to selected gram-negative bacteria including the major pathogens *Klebsiella pneumoniae* and *Pseudomonas aeruginosa* (19, 20, 21, 22, 23, 24). As a novel transmission and virulence factor, it promotes bacterial survival during temperature-, pressure-, and oxidant-based sterilization procedures applied in food production and in the hospital setting (19, 20, 25, 26, 27, 28, 29, 30), with subsequent establishment in the human gut (28, 31). *clpG* copies are typically located on mobile genomic islands and plasmids and are coorganized with additional ORFs encoding for protein quality control factors, including chaperones (small HSPs), proteases (FtsH, DegP, HtpX), or factors involved in oxidative stress response (thioredoxin) (23). The gene clusters (locus of heat resistance, transmissible locus for protein quality control) comprise up to 16 core ORFs (21, 22, 32). Notably, *clpG* can also be encoded in the core genome of bacteria, although this placement is less frequent (24). *P. aeruginosa* clone C strains encode for two *clpG* copies, one located in the core chromosome (*clpG*_*GC*_) and a second on the transmissible locus for protein quality control island (*clpG*_*GI*_). As a hallmark of the ClpG subfamily of Hsp100 disaggregases, both ClpG proteins exhibit stand-alone protein disaggregation activity *in vitro* and can functionally replace each other *in vivo*, indicating similar basic functionality irrespective of gene synteny (24).

GlpG and ClpB can functionally replace each other *in vivo* for basic lethal temperature tolerance (24, 33). However, the two disaggregases differ in fundamental mechanistic aspects as ClpB strictly requires cooperation with the Hsp70 (DnaK) chaperone system. These differences in mechanisms of disaggregation must be reflected in sequence variability of shared domains and/or the presence of extra domain(s); however, a systematic analysis of ClpG extra domain functions has not been performed. ClpG (ClpK) exhibits a similar core domain organization as compared with ClpB but possesses a distinct M domain and an N1 domain and a C-terminal extension (CTE) not present in other Hsp100 family members. Here, we dissect the roles of individual domains of *P. aeruginosa* ClpG_GI_, which represents the best characterized family member to date. By deleting individual extra domains, we confirm that solely the ClpG_GI_-specific N1 domain is essential for aggregate binding and subsequent disaggregation. Intriguingly, an N1 domain fusion converts ClpB into an autonomous disaggregase. ClpG_GI_ ATPase activity is negatively regulated by both N1 and N2 domains and positively by the ATPase activity of the alternate AAA+ domain, with substrate binding to override ATPase control by N1 and N2, directly linking substrate presence to high ATPase and disaggregation activities. Mutating pore-located aromatic residues in the AAA domains reduces (AAA1) or abolishes (AAA2) disaggregation activity, confirming substrate threading as a force-generating step in disaggregation and defining the AAA2 ring as a main threading motor. Together, these findings define the basic mechanism of ClpG-mediated disaggregation and ATPase control.

## Results

### Only the N1 domain of ClpG but no other extra domain is crucial for heat resistance and disaggregation activity

**Figure 1:**
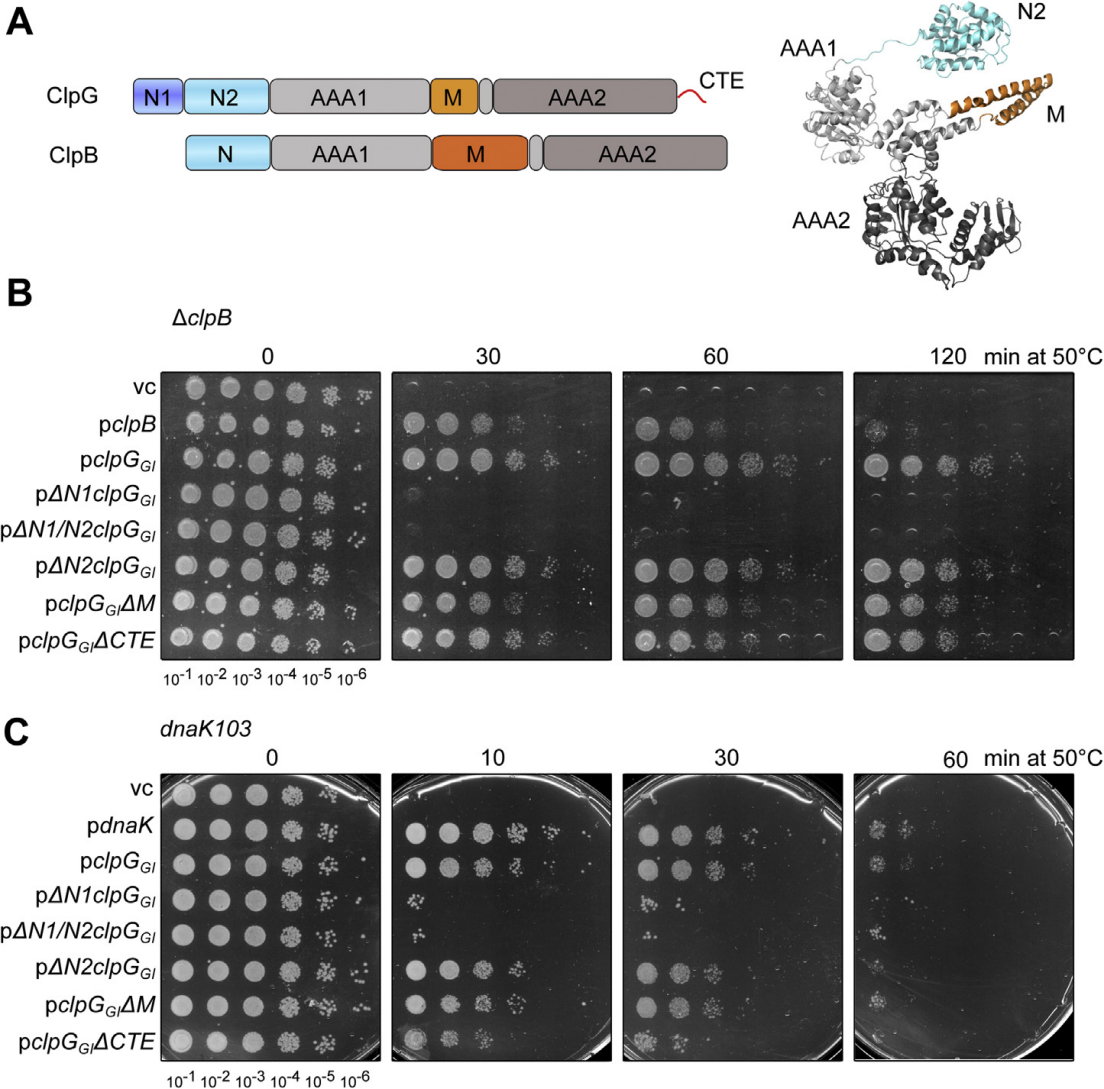
**The ClpG**_**GI**_**N1 domain is essential for heat resistance.***A*, domain organizations of ClpG and ClpB. Both Hsp100 proteins consist of two AAA domains (AAA1, AAA2), a middle domain (M) and a homologous N-terminal domain (ClpB: N, ClpG: N2). ClpG additionally harbors the N1 domain and a C-terminal extension CTE. A structural model of ClpG lacking N1 and CTE is provided. *B* and *C*, *E. coli* Δ*clpB* (*B*) or *dnaK103* (*C*) cells harboring plasmids for expression of *E. coli clpB*, *dnaK*, or *P. aeruginosa clpGGI* and its indicated deletion mutants (vc: empty vector control) were grown at 30 °C to midlogarithmic growth phase and shifted to 50 °C. Serial dilutions of cells were prepared at the indicated time points, spotted on LB plates and incubated at 30 °C. CTE, C-terminal extension; Hsp100, 100-kDa heat shock protein.

ClpG harbors two N domains (N1 and N2), two AAA domains (AAA1, AAA2), an AAA1-inserted M domain and a CTE (Fig. 1*A*, Fig. S1). Sequence conservations among ClpG homologs are very high in both AAA domains and lower in extra domains, as also observed for other Hsp100 family members (Fig. S2). The N1 domain represents a defining feature of ClpG proteins distinctively discriminating this subfamily from other Hsp100 proteins (Fig. 1*A*). The N2 domain of ClpG is homologous to N domains of other Hsp100 proteins such as ClpA, ClpB, and ClpC (Fig. 1*A*, Fig. S1). The ClpG M domain is predicted to form a coiled-coil structure composed of a single helical wing, like the M domain of ClpC but it is shorter than the ClpB M domain, which is composed of two wings (Fig. 1*A*, Fig. S1). ClpG also separates from ClpB by harboring a CTE including 20 to 40 residues that is typically enriched for proline and lysine residues and is predicted disordered.

The role of the N1 domain has been initially characterized. N1 deletion strongly reduces ClpG disaggregation activity *in vitro* and *in vivo* and can increase basal ATPase activity (24). The mechanistic basis of the crucial role of the N1 domain is not fully understood. N1 confers autonomous aggregate binding to *P. aeruginosa* ClpG_GC_ as a respective ΔN1 deletion mutant does no longer bind to protein aggregates. On the other hand, ΔN1–ClpG_GI_ still seems to interact with aggregated proteins *in vitro* (24). Therefore, it remained unclear whether the low disaggregation activity of ΔN1–ClpG stems from deficiencies in aggregate binding or is caused by indirect effects through, for example, deregulation of ATPase control.

To dissect the roles of ClpG characteristic domains, we focused our analysis on *P. aeruginosa* ClpG_GI_, which represents the best-characterized family member to date (24, 33) and generated ClpG_GI_ deletion mutants lacking individual domains. In addition, we deleted both N domains yielding ΔN1/2–ClpG_GI_. All deletion mutants were expressed in *Escherichia coli* Δ*clpB* and *dnaK103* mutant cells, which lack the ClpB/DnaK (Hsp70) disaggregase and are therefore sensitive to exposure to 50 °C. ClpG_GI_ can functionally replace ClpB and Hsp70 upon overexpression in *E. coli* and even provides increased heat resistance to Δ*clpB* cells as it exhibits higher disaggregation activity than the canonical ClpB disaggregase (24, 33). The production levels of ClpG_GI_ in complementation assays were similar to those of ClpB or DnaK controls produced from the same expression vector (Fig. S3*A*). ClpB or DnaK overexpression did not restore heat tolerance of *dnaK103* or *ΔclpB* cells, respectively, underlining the specific function of ClpG_GI_ as stand-alone disaggregase (Fig. S3, *B* and *C*). To test whether ClpG_GI_ overproduction is required for complementation activity, we expressed the disaggregase in *E. coli dnaK103* mutant cells in the presence of varying IPTG concentrations (0–250 μM) and determined production levels and cellular protection upon heat shock to 49 °C (Fig. S3, *D* and *E*). We observe that ClpG_GI_ overproduction in the presence of 100- to 250-μM IPTG is necessary for providing efficient heat resistance to these cells.

All ClpG_GI_-based deletion constructs except those lacking the N1 domain provided heat resistance to both *E. coli* mutant strains (Fig. 1, *B* and *C*), comparable with ClpG_GI_ WT except for ClpG_GI_-ΔCTE, which provided slightly reduced heat resistance to *dnaK103* cells. Expression levels of all mutant constructs were similar except for those lacking the N1 domain (Fig. S3, *F* and *G*), raising the possibility that lower levels of ΔN1–ClpG_GI_ and ΔN1/N2–ClpG_GI_ contribute to the lack of complementation activity *in vivo*.

We also tested the abilities of ClpG_GI_ deletion mutants to complement the temperature-sensitive growth phenotype of *E. coli dnaK103* cells at 41 °C (Fig. S4). We again find that the N1 domain is essential for complementation activity, while N2 or M domain deletions exhibited activities similar to ClpG_GI_ WT. A CTE deletion lowered the ability of ClpG_GI_ to complement significantly more than in the lethal heat tolerance assay (Fig. 1*C*). Notably, in this assay, the highest complementation activities were observed in presence of 25- to 50-μM IPTG, while higher IPTG concentrations particularly reduced the ability of ClpG_GI_ deletion mutants to restore growth at 41 °C. This finding suggests that high levels of these mutant proteins provoke deleterious effects in *dnaK103* cells at high temperatures.

**Figure 2:**
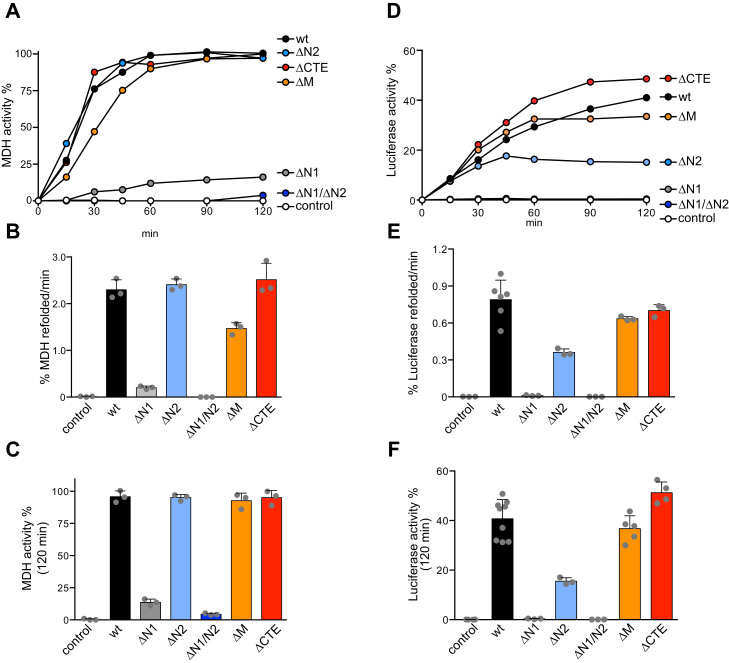
**The ClpG**_**GI**_**N1 domain is essential for protein disaggregation.***A*, disaggregation of aggregated malate dehydrogenase (MDH) by ClpG_GI_ WT or the indicated ClpG_GI_ deletion mutants was monitored by determining MDH activities at the indicated time point. The activity of native MDH was set at 100%. A control without ClpG is provided. *B* and *C*, MDH refolding rates (*B*) and reactivation yields (120 min) (*C*) were determined. SDs are based on three independent experiments. *D*, disaggregation of aggregated luciferase by ClpG_GI_ WT or the indicated ClpG_GI_ deletion mutants was monitored by determining luciferase activities at the indicated time point. The activity of luciferase before heat denaturation was set at 100%. *E* and *F*, luciferase refolding rates (*E*) and reactivation yields (120 min) (*F*) were determined. SDs are based on three independent experiments.

To better characterize and compare the ClpG_GI_ deletion mutants, we purified all constructs and determined their disaggregation activities toward heat-aggregated malate dehydrogenase (MDH) and firefly luciferase (Fig. 2). Deleting the N2 and M domains or the CTE still allowed for efficient reactivation of both aggregated model substrates by ClpG_GI_. ΔN2–ClpG_GI_ exhibited, however, reduced activity toward aggregated luciferase and reactivated 15.5% as compared with 40.7% by ClpG_GI_ WT (Fig. 2, *D* and *E*). Disaggregation kinetics toward luciferase aggregates were lower than aggregated MDH with lower renatured protein product, indicating that disentanglement of these aggregates is more challenging and less efficient. On the other hand, ΔN1–ClpG_GI_ did not reactivate aggregated Luciferase and exhibited 11.6-fold reduced disaggregation activity toward MDH as compared with ClpG_GI_ WT (Fig. 2, *A*–*C*). ΔN1/2–ClpG_GI_ did not exhibit any disaggregation activity, suggesting that the residual MDH disaggregation activity of ΔN1–ClpG_GI_ stems from the N2 domain. These results from *in vitro* disaggregation experiments largely agreed with those obtained from *in vivo* heat-resistance experiments. Together, these findings underline the most crucial role of the N1 domain for ClpG_GI_ disaggregation activity *in vitro* and *in vivo*, whereas all other extra domains are not essential.

### N1 fusion converts ClpB to an autonomous disaggregase

**Figure 3:**
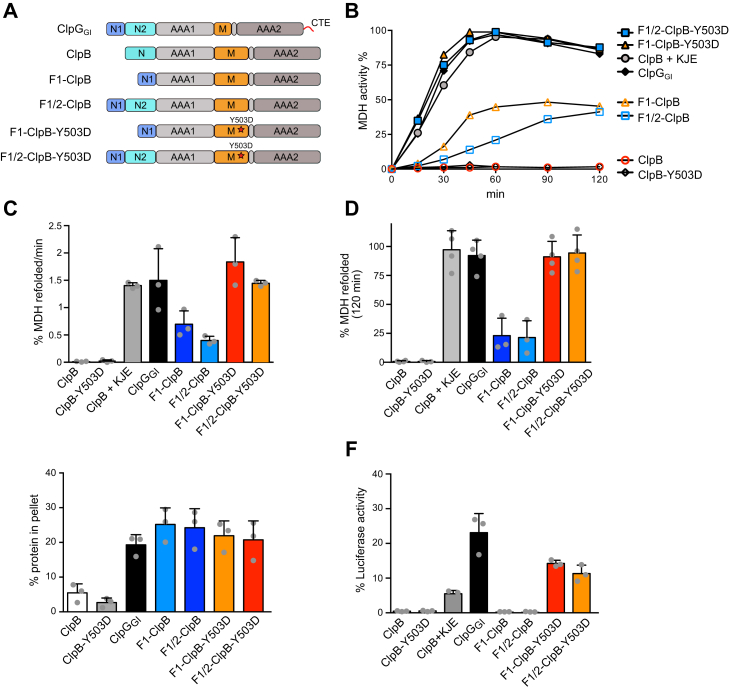
**Fusion of N1 domain converts ClpB into a stand-alone disaggregase.***A*, domain organizations of ClpG_GI_–ClpB chimeras. The ClpBM-domain mutation Y503D abrogates repression of the ClpB ATPase motor. *B*, disaggregation of aggregated malate dehydrogenase (MDH) by ClpG_GI_ WT, ClpB, ClpB–Y503D, ClpB with the DnaK chaperone system (KJE), or indicated ClpG_GI_–ClpB chimeras was monitored by determining MDH activities at the indicated time point. The activity of native MDH was set at 100%. *C* and *D*, MDH refolding rates (*C*) and reactivation yields (120 min) (*D*) were determined. SDs are based on three independent experiments. *E*, heat-aggregated MDH was incubated with the indicated Hsp100 proteins in the presence of 2-mM ATPgS. Soluble and insoluble (pellet) fractions were separated by centrifugation and analyzed by SDS-PAGE. Intensities of protein bands were quantified by ImageJ, and the fraction (%) of Hsp100 protein present in the pellet fraction was determined. The fractions (%) of Hsp100 proteins present in the pellet of control reactions (lacking MDH aggregations) were subtracted. SDs are based on three independent experiments. *F*, disaggregation of aggregated luciferase by ClpG_GI_ WT, ClpB, ClpB-Y503D, ClpB with the DnaK chaperone system (KJE) or indicated ClpG_GI_–ClpB chimeras was monitored by determining luciferase activities. The activity of native luciferase was set at 100%. Luciferase reactivation yields (90 min) are shown. SDs are based on three independent experiments. Hsp100, 100-kDa heat shock protein.

We were wondering whether we could overcome ClpB dependence on DnaK and converting it into a stand-alone disaggregase by fusing N1 or N1/N2 domains of ClpG_GI_ to the ClpB ATPase motor composed of its AAA1 and AAA2 domains and the regulatory M domain (Fig. 3*A*). F1–ClpB and F1/2–ClpB showed partial disaggregation activity and reactivated 20.1% and 18.2% of MDH after 120 min, respectively. Because DnaK also activates the ClpB ATPase ring by displacing repressing M domains, we additionally generated chimeras harboring the M domain mutation Y503D, which causes derepression of the AAA domains independent of DnaK (16, 34). Purified fusion constructs were probed for reactivation of aggregated MDH. F1–ClpB–Y503D and F1/2–ClpB–Y503D exhibited MDH refolding kinetics and yields that were indistinguishable from ClpG_GI_ or ClpB in the presence of the DnaK chaperone system (KJE: DnaK, DnaJ, GrpE). These findings imply that fusion of N1 and concurrent abolition of M domain repression converts ClpB into a highly efficient and stand-alone disaggregase. Accordingly, the disaggregation activity of F1–ClpB could be increased upon addition of DnaK507 (Fig. S4, *A*–*C*). DnaK507 lacks the C-terminal helical bundle of the DnaK substrate binding domain and activates ClpB ATPase activity by binding and displacing M domains (35). In absence of its J domain protein partner, DnaJ, DnaK507 cannot bind to substrates and thus does not recruit ClpB to protein aggregates. Accordingly, DnaK507 did not allow for ClpB-mediated protein disaggregation (Fig. S5, *A*–*C*). The stimulatory effect of DnaK507 on F1–ClpB therefore only involves the displacement of repressing M domains and demands for autonomous binding of F1–ClpB to the aggregated substrate. To directly demonstrate that N1 fusion enables ClpB to bind to protein aggregates, we determined the binding capacities of all fusion constructs and respective ClpB and ClpG_GI_ controls (Fig. 3*E*, Fig. S5*D*). All fusion constructs bound to aggregated MDH with similar efficiencies, demonstrating that (i) N1 fusion confers aggregate binding capacity and (ii) increased disaggregation activities of F1–ClpB–Y503D and F1/2–ClpB–Y503D solely stem from a derepressed ATPase motor. We then probed all constructs for disaggregation of heat-aggregated luciferase as an alternative model substrate. Here, only F1–ClpB–Y503D and F1/2–ClpB–Y503D exhibited disaggregation activity (Fig. 3*F*, Fig. S5*E*). This confirms the previous observation that the disaggregation of luciferase aggregates demands for higher force application and, accordingly, disaggregation by ClpG_GI_–ClpB chimeras is only observed upon additional derepression of ClpB ATPase activity by M-domain mutation. In agreement with such scenario, ClpG_GI_ was 4.2-fold more efficient in luciferase reactivation than ClpB/KJE, consistent with our former finding that ClpG_GI_ is a more potent disaggregase (33). We infer that fusion of N1 is sufficient, enabling ClpB to directly bind aggregated proteins. The additional fusion of the N2 domain does not further increase disaggregation activity of the fusion constructs, indicating that N2 plays an inferior role in ClpG_GI_-mediated disaggregation.

### Both AAA domains are crucial for protein disaggregation

**Figure 4:**
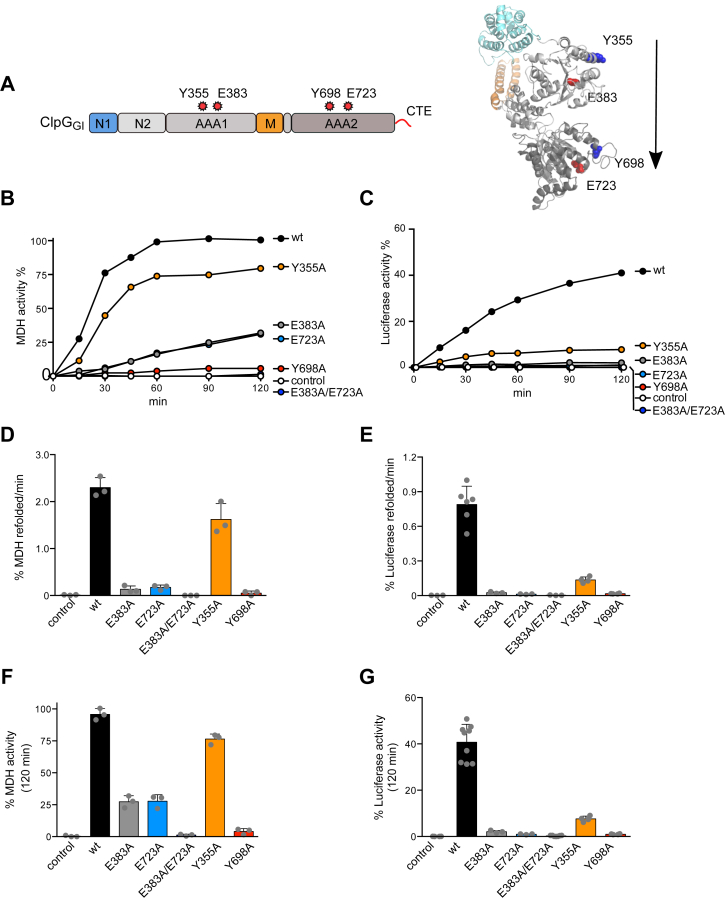
**ATP-driven substrate threading is propelling ClpG**_**GI**_**protein disaggregation.***A*, domain organization and structural model of ClpG_GI_ (lacking N1 and CTE). Positions of Walker B (E383, E723) and pore loop (Y355, Y698) residues are indicated. The relative position and directionality of the ClpG_GI_ translocation channel is indicated by an *arrow*. *B*, disaggregation of aggregated malate dehydrogenase (MDH) by ClpG_GI_ WT or indicated mutants was monitored by determining MDH activities at the indicated time points. A control without Hsp100 addition is provided. The activity of native MDH was set at 100%. *C* and *D*, MDH refolding rates (*C*) and reactivation yields (120 min) (*D*) were determined. *E*, disaggregation of aggregated luciferase by ClpG_GI_ WT or the indicated mutants was monitored by determining luciferase activities at the indicated time points. A control without Hsp100 addition is provided. The activity of native luciferase was set at 100%. *F* and *G*, luciferase refolding rates (*F*) and reactivation yields (120 min) (*G*) were determined. SDs are based on three independent experiments. CTE, C-terminal extension; Hsp100, 100-kDa heat shock protein.

We next sought to dissect the roles of the individual AAA domains (AAA1, AAA2), which form the ATP-driven threading motor, in ClpG_GI_ disaggregation. Here, we abolished ATP turnover in individual AAA domains by mutating the glutamate residues essential for ATP hydrolysis of the conserved Walker B hhDE motifs E383A (AAA1) and E723A (AAA2). Disaggregation activities of ClpG_GI_ ATPase mutants were determined using MDH and luciferase aggregates as before. Both single Walker B mutants showed minor disaggregation activities toward aggregated MDH that were 20.8-fold (E383A) and 14.2-fold (E723A) reduced as compared with ClpG_GI_ WT (Fig. 4, *B*–*D*). These residual activities further decreased when using aggregated luciferase as a substrate, and only very low reactivation yields were determined after 120 min (2% for E383A, 1% for E723A as compared with 40.7% for ClpG_GI_ WT) (Fig. 4, *E*–*G*). Mutating both Walker B motifs (E383A/E723A) entirely abrogated protein disaggregation.

ATP hydrolysis in each AAA domain propels changes in positions of conserved, pore-located aromatic residues, which bind and pull substrates through the central translocation channel (Fig. 4*A*). We generated respective ClpG_GI_ pore loop mutants (Y355A (AAA1), Y698A (AAA2)), which are predicted to be affected in substrate threading at either ATPase ring. ClpG_GI_–Y355A retained substantial activity toward aggregated MDH, although it was 5.5-fold less efficient than ClpG_GI_ WT in case of aggregated luciferase (Fig. 4). ClpG_GI_-Y698A did not exhibit any disaggregation activity toward both model substrates in contrast to ClpG_GI_-Y355A. This indicates that substrate threading is an essential part of ClpG_GI_-mediated protein disaggregation with most important contributions by pore-located residues of the AAA2 ring.

### N1 and N2 domains repress ClpG ATPase activity

**Figure 5:**
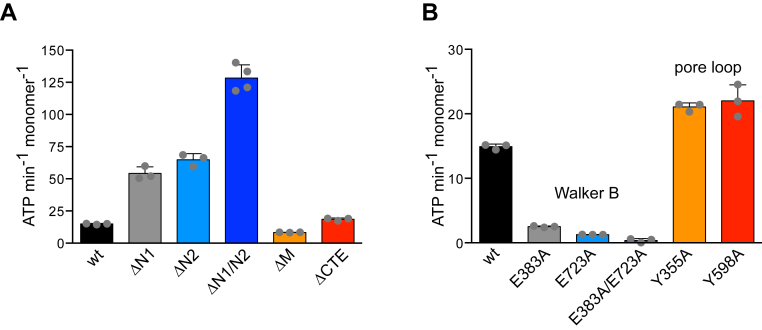
**N1 and N2 domains repress ClpG**_**GI**_**ATPase activity.***A* and *B*, ATPase activities of ClpG_GI_ and the indicated deletion (*A*) or point mutants (*B*) were determined. SDs are based on three independent experiments.

We probed for functions of extra domains in regulating ClpG_GI_ ATPase activity. Consistent with our initial report, ClpG_GI_ has a high basal ATPase activity (15 ATP min^−1^ monomer^−1^) as compared with the canonical disaggregase ClpB (1.7 ATP min^−1^ monomer^−1^), consistent with our initial report (24). Deleting either N1 or N2 caused a 4-fold increase in ATP hydrolysis rates (Fig. 5*A*). ΔN1/N2–ClpG_GI_ exhibited very high ATPase activity (128 ATP min^−1^ monomer^−1^), which was slightly higher than the sum of ATPase activities of the respective single deletion constructs (equals 104 ATP min^−1^ monomer^−1^). This indicates that both N domains individually and synergistically repress ClpG_GI_ ATPase activity. Notably, the very high ATPase activity of ΔN1/N2–ClpG_GI_ is similar to the fully activated ATPase motor of ClpB, once ClpB is recruited to an aggregated protein by DnaK (36). The basal ATPase activity of ClpG_GI_–ΔCTE was similar to ClpG_GI_ wt, whereas ΔM–ClpG_GI_ exhibited 1.8-fold reduced activity (Fig. 5*A*). We speculated that this might be caused by decreased stability of ΔM–ClpG_GI_ oligomers, as such defect was reported previously for ClpB M domain deletion mutants (37). We monitored the ATP-dependent oligomerization of ClpG_GI_ by size-exclusion chromatography (SEC) (Fig. S6). In the absence of ATP, ClpG_GI_ eluted as monomer, while nucleotide presence triggered oligomerization. We observed similar elution patterns for ΔN1–ClpG_GI_, ΔN2–ClpG_GI_ and ClpG_GI_-ΔCTE, whereas ΔM–ClpG_GI_ eluted as monomer. This suggests that ΔM–ClpG_GI_ oligomers are less stable and dissociate during the SEC run. Notably, ΔN1/N2–ClpG_GI_ also did not elute as oligomer in contrast to N1 and N2 single deletion constructs. We speculated that the very high ATPase activity of ΔN1/N2–ClpG_GI_ might destabilize ΔN1/N2–ClpG_GI_ oligomers. We therefore aimed at inhibiting ATP hydrolysis by mutating the Walker B motifs of both AAA domains (E383A/E723A, see below). ΔN1/N2–ClpG_GI_–E383A/E723 was not affected in ATP-driven oligomer formation, indicating that oligomerization does not involve N domains (Fig. S6).

We next determined the individual contributions of the AAA1 and AAA2 domains to ClpG_GI_ ATPase activity and whether they cooperate during the ATPase cycle by analyzing single Walker B mutants (E383A, E723A), which do not hydrolyze ATP at the AAA1 or AAA2 ring. We observed that the ATPase activities of both mutants were strongly reduced by 6- and 12-fold (E383A and E723A, respectively) (Fig. 5*B*). ClpG_GI_-E383A/E723A was deficient in ATP hydrolysis, confirming that the individual mutations abrogate ATPase activity in each AAA domain. The sum of ClpG_GI_–E383A and ClpG_GI_–E723A ATPase activities was much lower than that of ClpG_GI_ WT (4 *versus* 15 ATP/min/monomer), indicating cooperation between both AAA rings during the ATPase cycle. ATP-induced oligomerization of all Walker B mutants was similar to ClpG_GI_ WT, excluding structural defects as a basis of reduced ATPase activities (Fig. S5). Similarly, the ClpG_GI_ pore loop mutants (Y355, Y698A) formed oligomers in the presence of ATP and exhibited slightly (1.4-fold) increased basal ATPase activities (Fig. 5*B*, Fig. S5). This demonstrates that the defects of pore loop mutants in protein disaggregation are directly caused by deficiencies in substrate threading.

### N1 binding peptide strongly stimulates ClpG_GI_ ATPase activity

**Figure 6:**
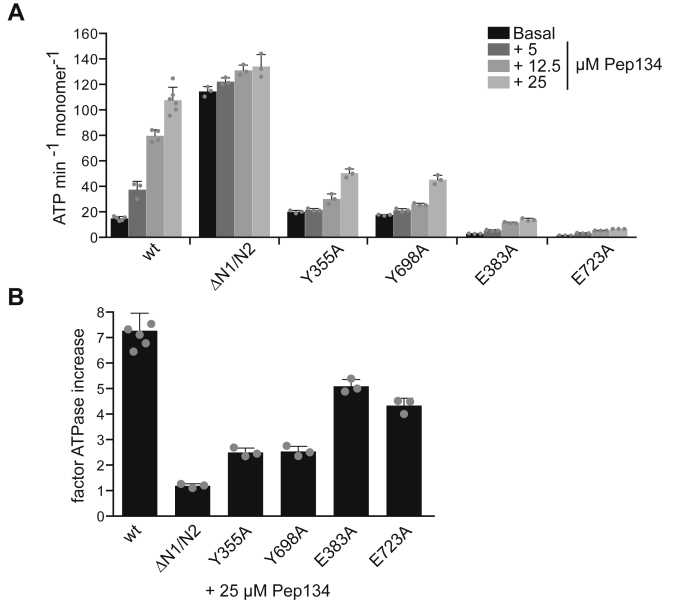
**A peptide substrate strongly increases ClpG**_**GI**_**ATPase activity.***A*, ATPase activities of ClpG_GI_, ΔN1/N2–ClpG_GI_, and the indicated point mutants were determined in the presence of increasing Pep134 concentrations. *B*, the factor of ATPase stimulation by 25-μM Pept134 was determined for ClpG_GI_ and indicated mutants. The basal ATPase activities were separately set at 1 for each protein. SDs are based on three independent experiments.

The observation that ΔN1/N2–ClpG_GI_ exhibits a derepressed, high ATPase activity raises the possibility that substrate binding to N1 and N2 triggers ATP hydrolysis. Because aggregated proteins cannot be added in excess over ClpG_GI_ in ATPase measurements, we sought to identify peptides that are bound by the N1 domain. We therefore determined the binding pattern of N1 to a peptide library consisting of 13-mer peptides overlapping by 10 residues and jointly representing the sequence of *S. cerevisiae* Has1, which served before as a model protein determining the binding specificity of Hsp70 chaperones (38). We observed binding of N1 to multiple Has1 peptides, which were typically enriched for aliphatic (I, L) and aromatic (F, Y) residues and disenriched for acidic (D, E) ones (Fig. S7, *A* and *B*). These features resemble the composition of peptides interacting with DnaK (39), explaining why ClpG_GI_ and DnaK compete for binding to protein aggregates. The interacting peptide 134 (FLRYLKASKVPLN) strongly increased the ATPase activity of ClpG_GI_ by 7.3-fold to 108 ATP min^−1^ monomer^−1^ with saturation of the stimulation at 25-μM peptide (Fig. S7*C*). In contrast, the nonbinding peptide 2 (SNKRSRDSESTEE) did not increase ATPase activity (Fig. S7*D*). Peptide 92 (KRFLLLFSFLKRN), which is also strongly bound by N1, could not be tested because of poor solubility. Notably, the Pep134-stimulated ClpG_GI_ ATPase activity is similar to the basal ATPase activity of ΔN1/N2–ClpG_GI_ (128 ATP min^−1^ monomer^−1^), implying that peptide binding indeed almost entirely abrogates repression by the N-domains. Accordingly, the high basal ATPase activity of ΔN1/N2–ClpG_GI_ was only weakly stimulated by Pep134, suggesting that peptide binding to N1 and N2 triggers ATP hydrolysis (Fig. 6). The residual increase in ΔN1/N2–ClpG_GI_ likely involves peptide binding to the ClpG_GI_ translocation channel, as ATPase stimulation of the pore loop mutants Y355A and Y698A was reduced (Fig. 6). Analysis of the single Walker B mutants E383A and E723A revealed that both ATPase rings are stimulated by Pep134, although the total ATPase activity remained low, confirming that two functional AAA domains are required for high ATPase activity (Fig. 6).

## Discussion

In the presented study, we dissected the basic mechanism of the stand-alone ClpG disaggregase and compared it with the Hsp70-dependent canonical ClpB disaggregase. We mainly focused on the functions of ClpG extra domains, as those provide functional diversity to AAA+ proteins. We show that the ClpG-specific N1 domain but no other extra domain is essential for disaggregation *in vitro* and *in vivo*. This unique function of N1 is based on its role in conferring autonomous aggregate binding capacity to ClpG, explaining why ClpG does not require a partner for substrate interaction unlike ClpB and ClpC. Transplanting N1 onto ClpB confers Hsp70-independent binding to protein aggregates and high disaggregation activity upon additional abolition of M-domain repression. These finding clarify why ΔN1–ClpG_GI_ is deficient in disaggregation. We assume that the previously noticed binding of ΔN1–ClpG_GI_ to aggregated luciferase *in vitro* (24) is unspecific and nonproductive and largely caused by the sticky nature of this construct.

Roles for other extra domains in ClpG-mediated disaggregation are currently not well defined. The N2 domain contributes to disaggregation power, as (i) its deletion reduces reactivation efficiency of aggregated luciferase (Fig. 2, *D* and *E*) and (ii) ΔN1–ClpG_GI_ but not ΔN1/N2–ClpG_GI_ exhibits residual disaggregation activity toward aggregated MDH (Fig. 2, *A*–*C*). The N2 domain is homologous to N-domains of ClpB and ClpC. The ClpB N domain is not essential for protein disaggregation; however, its deletion reduces disaggregation efficiency in a substrate-specific manner (40, 41, 42). Its contribution is explained by the presence of a hydrophobic groove that binds soluble misfolded proteins, likely enhancing substrate gripping during the threading and disaggregation process (42, 43). Key hydrophobic residues forming the substrate binding site of the ClpB N domain are also present in ClpG_GI_ N2 (I112/I120/L193/Y207). We therefore speculate that the N2 domain offers additional binding sites for substrates, explaining its contribution to ClpG_GI_-mediated disaggregation. Disaggregation activities of ClpG_GI_ mutants lacking either the M domain or CTE hardly differ *in vitro* from ClpG_GI_ WT. The ClpB M domain is essential for disaggregation as it serves as the binding platform for the Hsp70 partner. The autonomous disaggregation activity of ClpG, mediated by N1, rationalizes why ΔM–ClpG_GI_ is functional *in vivo* and *in vitro*. ClpG_GI_–ΔCTE displayed reduced complementation activity in *E. coli dnaK103* mutant cells, pointing to a specific requirement for the CTE *in vivo*. Yet, it has to be tested whether similar results are obtained when testing ClpG_GI_–ΔCTE activity in *P. aeruginosa* cells. The roles of the ClpG_GI_ M domain, N2 domain, and CTE require further investigation. The analysis presented here is based on *in vitro* work and complementation studies in *E. coli* mutant cells. We therefore cannot rule out specific functions of these extra domains in regulating ClpG_GI_ function in *P. aeruginosa* cells.

Mutating key residues crucial for ATP hydrolysis and substrate threading in both AAA rings revealed that ClpG exerts an ATP-fueled threading activity like ClpB. ATP hydrolysis in both AAA domains is crucial for disaggregation. However, the AAA domains differ with respect to the roles of conserved pore loop residues in substrate threading. The AAA1 pore loop mutant Y355A still retains substantial disaggregation activity, whereas the respective AAA2 mutant Y698A is inactive. This difference in pore loop contributions is analogous to ClpB, indicating that substrate gripping by pore loops of the AAA2 ring is in general most relevant for protein disaggregation. We speculate that additional substrate contacts to, for example, the adjacent N2 domain might partially compensate for the loss of the AAA1 pore loop residue, as has been suggested for ClpB (41, 44).

When analyzing ATPase activity control of ClpG_GI_, we noticed not only similarities but also substantial differences to ClpB. The basal ATPase activity of ClpG_GI_ is strongly increased in comparison to ClpB (15 *versus* 1.7 ATP min^−1^ monomer^−1^) consistent with former findings (24). This increase is, however, not sufficient to mechanistically explain ClpG_GI_ autonomous disaggregation activity. ClpB ATPase activity during protein disaggregation is strongly increased by Hsp70 and substrate interaction to 120 ATP min^−1^ monomer^−1^ (36), a value much higher than the basal ATP hydrolysis rate of ClpG_GI_. This value is similar to the strongly increased basal ATPase activity (128 ATP min^−1^ monomer^−1^) of ΔN1/N2–ClpG_GI_ (Fig. 5*A*). This finding indicates that N1 and N2 domains repress ClpG_GI_ ATPase activity and predicts that substrates stimulate ATP hydrolysis through binding to N1 and N2 domains, overriding the repressive functions of the extra N domains in ATPase control. Indeed, an N1 binding peptide strongly stimulates ClpG_GI_ ATPase activity to 108 ATP/min/monomer (Fig. 6), a value comparable to fully activated ClpB. We therefore suggest that both disaggregases can reach similar ATPase rates during protein disaggregation. High ATPase activities of ClpB are likely restricted to the initial phase of disaggregation and drop upon Hsp70 dissociation and redocking of M domains onto the AAA1 ring (36, 45). This mechanistically explains why ClpB does not exhibit high unfolding power in contrast to other Hsp100 proteins and cannot unfold stable domains during protein disaggregation (46). Disaggregation and unfolding activities of ClpG_GI_ are higher than those of ClpB (33), indicating persistent stimulation of ClpG_GI_ ATPase by substrates.

Both AAA domains of ClpG_GI_ communicate during the ATPase cycle, and the cooperation seems different as compared with ClpB. ATP hydrolysis in the ClpB AAA1 and AAA2 domains is anticorrelated, and blocking ATP hydrolysis in one AAA ring is increasing ATPase activity in the remaining functional AAA ring (36, 47). In contrast, mutationally abolishing ATP hydrolysis at either AAA1 or AAA2 of ClpG_GI_ by single Walker B mutants (E383A, E723A) strongly reduces the basal ATPase activity of the intact AAA domain. This indicates positive cooperation between the ClpG_GI_ ATPase rings upon hydrolysis. How exactly ATP binding and hydrolysis events in one AAA ring are signaled to the other AAA ring is unknown and demands further investigation.

We conclude that ClpG_GI_ disaggregation activity is based on an ATP-fueled threading activity. Binding of ClpG_GI_ to protein aggregates is mediated by its unique N1 domain and triggers ATP hydrolysis by overriding ATPase repression by N1 and N2 domains. ClpG_GI_ ATPase activity is thereby directly controlled by substrate presence and does not involve additional factors in contrast to Hsp70-dependent ClpB.

## Experimental procedures

### Strains and plasmids

All strains and plasmids used in this study are summarized in Table S1. *E. coli* cells were grown in LB medium containing appropriate antibiotics at 30 °C with an agitating speed of 120 rpm. *E. coli* XL1 blue was used for cloning and retaining of plasmids requiring kanamycin (Km) at 30 μg ml^−1^ and ampicillin (Ap) at 100 μg ml^−1^ for plasmid propagation.

### Protein purification

*E. coli* ClpB was purified after overproduction from *E. coli* Δ*clpB::kan* cells using pDS56-derived expression vectors (16). *P. aeruginosa* ClpG_GI_ and mutant derivatives and DnaK507 were purified after overproduction in *E. coli* BL21 cells using pET24a-derived expression vectors. ClpG_GI_ deletion mutants and ClpG_GI_/ClpB hybrids were generated by PCR, and point mutants were constructed by QuickChange one-step site-directed mutagenesis. All mutations were verified by sequencing. All proteins harbor a C-terminal His_6_-tag and were purified using Ni-IDA (Macherey-Nagel) following the instructions provided by the manufacturer. In short, cell pellets were resuspended in buffer A (50-mM NaH_2_PO_4_, 300-mM NaCl, 5-mM β-mercaptoethanol, pH 8.0) supplemented with protease inhibitors (Roche). After cell lysis using French press, the cell debris was removed by centrifugation at 16,000*g* for 45 min at 4 °C, and the cleared lysate was incubated with Protino IDA resin (Macherey-Nagel) for 1 h at 4 °C. Afterward, the resin was transferred into a plastic column and washed once with buffer A. His-tagged proteins were eluted by addition of buffer A supplemented with 250-mM imidazole. Subsequently, pooled protein fractions were subjected to SEC (Superdex S200, Amersham) in buffer A1 (50-mM Tris, pH 7.5, 50-mM KCl, 10-mM MgCl_2_, 5% (v/v) glycerol, 2-mM DTT) for ClpB and buffer A2 (50-mM Tris, pH 7.5, 150-mM KCl, 5% (v/v) glycerol, 2-mM DTT) for ClpG.

Purification of C-terminal Twin-Strep-Tag tagged N1–domain–ClpG_GI_ was performed with Strep-Tactin Superflow High Capacity resin (IBA Lifesciences) as described by the manufacturer. Afterward, the purified protein was subjected to gel filtration using a Superdex S30 (GE Healthcare) in buffer B (100-mM Tris-HCl, 150-mM NaCl, 5% (v/v) glycerol, 2-mM DTT, pH 7.5).

Purifications of DnaK, DnaJ, GrpE, and firefly luciferase were performed as described previously (11, 16, 46). Pyruvate kinase of rabbit muscle and MDH of pig heart muscle were purchased from Sigma. Protein concentrations were determined with the Bradford assay (Bio-Rad).

### Heat-resistance assays

*E. coli ΔclpB* or *dnaK103* cells harboring pUHE21 derivates allowing for IPTG-controlled expression of *clpB*, *clpG*_*GI*_ (WT or mutants), or *dnaK* were grown in LB media at 30 °C to early logarithmic phase (optical density at 600 nm: 0.15–0.2). Expression of the respective proteins was induced by addition of IPTG (*clpB*: 20-μM IPTG, *clpG*_*GI*_, *dnaK*: 100-μM IPTG). Protein production was documented 2 h after IPTG addition by Western blot analysis. Subsequently, 1-ml aliquots were shifted to 50 °C for 60 (*dnaK10*3) or 120 (*ΔclpB*) min. At indicated time points, bacterial survival was determined by preparing serial dilutions, spotting them on LB plates followed by incubation for 24 h at 30 °C.

### Complementation of *E. coli dnaK103* temperature sensitivity

Serial dilutions of overnight cultures of *E. coli dnaK103* cells harboring pUHE21 derivates allowing for IPTG-controlled expression of *clpG*_*GI*_ (WT or mutants) were spotted on LB plates including varying IPTG concentrations. Plates were incubated at 30 °C or 41 °C for 24 h, and colony formation was determined.

### Bioinformatic analysis

Multiple sequence alignments were performed using Clustal Omega (https://www.ebi.ac.uk/Tools/msa/clustalo/) and were displayed using Jalview (http://www.jalview.org). An incomplete 3D model of ClpG_GI_ was generated by SWISS-MODEL (https://swissmodel.expasy.org) (48) using pdb-file 3j3r.1.G as template (*B. subtilis* ClpC).

### *In vitro* disaggregation assays

2 μM MDH (Roche) or 200-nM firefly luciferase was heat-aggregated at 47 °C for 30 min or 46 °C for 15 min, respectively, in assay the buffer (50-mM Tris, pH 7.5, 50-mM KCl, 20-mM MgCl_2_, 2-mM DTT). Aggregated proteins were mixed 1:1 with disaggregases (final concentrations: 1-μM ClpG (WT or mutants) or 1-μM ClpB (WT or mutants) with 1-μM DnaK, 0.2-μM DnaJ, 0.1-μM GrpE (KJE) or with 2-μM DnaK507)). The disaggregation reaction was started by addition of an ATP-regenerating system (2-mM ATP, 3-mM phosphoenolpyruvate, 20 ng/μl pyruvate kinase) and performed at 30 °C in the assay buffer. MDH activity was determined at indicated time points by measuring ΔA_340 nm_/min in a photometer. Here, 10 μl of disaggregation reaction was mixed with 690 μl of MDH assay buffer (150-mM potassium phosphate, 0.5-mM oxaloacetate, 0.28-mM NADH, 2-mM DTT, pH 7.6). Luciferase activities were determined using a Lumat LB 9507 (Berthold Technologies). 2 μl of disaggregation reaction was mixed with 125-μl luciferase assay buffer (25-mM glycylglycine, pH 7.4, 12.5-mM MgSO_4_, 5-mM ATP) and subsequently 125-μl luciferin (Gold Biotechnology) were injected. 100% activity corresponds to MDH or luciferase activity before heat denaturation. Disaggregation activities were quantified based on initial disaggregates rates.

### ATPase assay

The ATPase activities of 1-μM ClpG WT or mutants were determined in the presence or absence of Pep134 in a reaction volume of 100 μl in the assay buffer with 0.5-mM NADH (Sigma), 1-mM PEP (Sigma), and 1/100 (v/v) PK/LDH mix (Sigma). 100 μl of 4-mM ATP in the assay buffer (50-mM Tris, pH 7.5, 50-mM KCl, 20-mM MgCl_2_, 2-mM DTT) was added to each reaction in a 96-well plate (TPP) format to start the reaction. The decrease of NADH absorbance at 340 nm was determined in a BMG Labtech FLUOstar Omega plate reader at 30 °C. ATPase activities were calculated by assuming a 1:1 stoichiometry of NAD^+^ oxidation and the production of ADP.

### Protein aggregate binding assay

To examine the interaction between ClpB, ClpG_GI_, or hybrid Hsp100 proteins and protein aggregates, 4-μM MDH was heat-denatured at 47 °C for 30 min in the assay buffer (50-mM Tris, pH 7.5, 50-mM KCl, 20-mM MgCl_2_, 2-mM DTT). MDH aggregates were mixed with 1.5-μM ClpB/ClpG_GI_/hybrid proteins and 2 mM of ATPγS in 100-μl assay buffer and incubated at 25 °C for 10 min. Soluble and insoluble fractions were separated by centrifugation at 13,000 rpm for 25 min at 4 °C. The pellet fraction was washed once with 150-μl assay buffer and centrifuged again at 13,000 rpm for 10 min at 4 °C. Binding assays were performed in Low Binding Micro Tubes (Sarstedt). The supernatant and pellet fractions were mixed with the protein sample buffer and analyzed by Coomassie staining after SDS-PAGE (8–16% gradient gels). Each assay was repeated three times independently. As control, purified Hsp100 proteins without aggregated proteins were subjected to the same protocol. Band intensities were analyzed using ImageJ.

### Peptide library screening

A peptide library membrane (PepSpots) composed of 13-mer peptides representing the primary sequence of *S. cerevisiae* Has1 was first activated in methanol for 10 min at room temperature (RT) and washed three times in Tris-buffered saline (TBS; 31-mM Tris–HCl, 170-mM NaCl, 6.4-mM KCl, pH 7.6) and once in buffer C (10-mM Tris, pH 7.5, 150-mM KCl, 20-mM MgCl_2_, 5% (w/v) sucrose, 0.005% (v/v) Tween 20). Next the membrane was incubated with 2-μM purified ClpG_GI_ N1 domain for 30 min at RT while gentle shaking. The membrane was washed for 15 s in ice-cold TBS, and the peptide-bound ClpG_GI_ N1 domain was electrotransferred onto a polyvinylidene difluoride (PVDF) membrane that was previously activated in methanol. For the electrotransfer, the blotting stack was packed as follows from the cathode to the anode: Whatman paper soaked in the buffer (57.7-mM Tris HCl, 2.1 M 6-amino hexanoic acid, 0.1% SDS), peptide library, activated PVDF membrane, Whatman paper soaked in the buffer (69.2-mM Tris HCl), and Whatman paper soaked in the buffer (231-mM Tris HCl). The transfer was performed using a semidry blotter at constant power transfer of 0.8 mA/cm^2^ for 30 min at 4 °C. The transfer was repeated three more times using a new PVDF membrane each time. Transferred ClpG_GI_ N1 domain was detected using ClpG_GI_ N1 domain–specific polyclonal antibodies (rabbit, 1:10,000) and an anti-rabbit alkaline phosphatase conjugate (Vector Laboratories) was used as the secondary antibody (1:20,000). Polyclonal antibodies were raised against purified N1 of ClpG_GI_ in rabbits by Davids Biotechnologie GmbH.

### Western blotting

Total extracts of cells were prepared and separated by SDS-PAGEs, which were subsequently electrotransferred onto a PVDF membrane. The membrane was incubated in the blocking solution (3% bovine serum albumin (w/v) in TBS) for 1 h at RT. Protein levels were determined by incubating the membrane with ClpG_GI_-specific antibodies (1:10,000 in TBS-T + 3% (w/v) bovine serum albumin) and an anti-rabbit alkaline phosphatase conjugate (Vector Laboratories) as the secondary antibody (1:20,000). Blots were developed using ECF Substrate (GE Healthcare) as the reagent and imaged *via* Image-Reader LAS-4000 (Fujifilm). Band intensities were quantified with ImageJ.

### SEC

Oligomerization of ClpG_GI_ WT and mutants was monitored by SEC using a Superdex S200 10/300 column (GE Healthcare) at 4 °C. The column was equilibrated with the assay buffer (±2 mM ATP). 10 μM ClpG_GI_ (WT or mutant) was preincubated in the assay buffer ±2-mM ATP for 5 min at RT before injection. Elution fractions were analyzed by SDS-PAGE followed by SYPRO staining. Thyroglobulin and γ-globulin served as molecular mass standards. SYPRO Ruby Protein Gel Stain (Molecular Probes) was performed as instructed by the manufacturer.

## Data availability

All data are contained within the article.

## Supporting information

This article contains supporting information (16, 24, 49, 50).

## Conflict of interest

No author has an actual or perceived conflict of interest with the contents of this article.
