# Supplementary material for: Basic mechanism of the autonomous ClpG disaggregase
Source: J Biol Chem. 2021 Feb 24;296:100460. doi: 10.1016/j.jbc.2021.100460 (PMC8024975; doi:10.1016/j.jbc.2021.100460)
Supplement: Supplemental Figures S1–S6 and Table S1 [file mmc1.pdf]

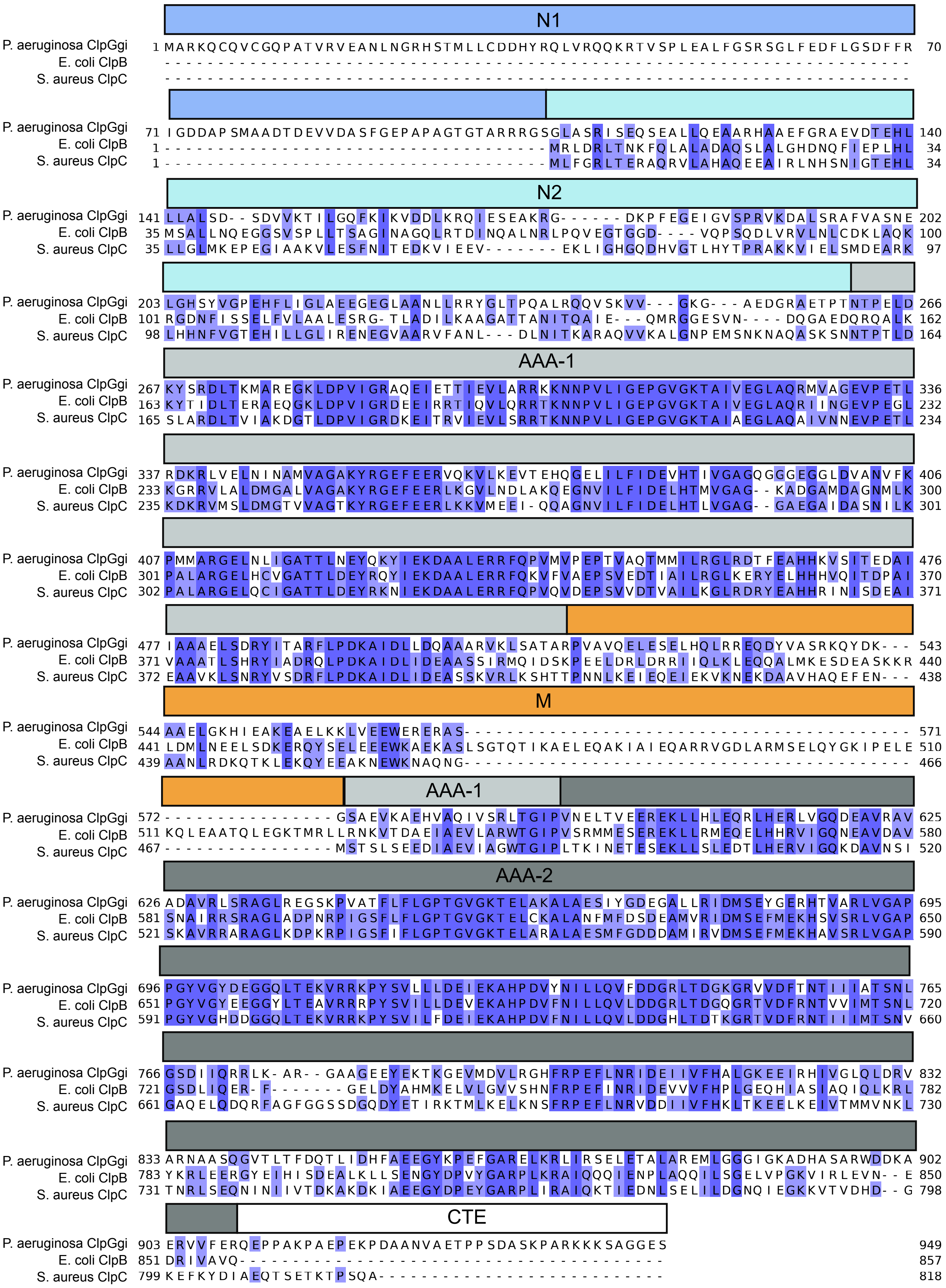

Figure S1

Sequence alignment of *Pseudomonas aeruginosa* ClpG$_{GI}$, *Escherichia coli* ClpB and *Staphylococcus aureus* ClpC. The domain organization is indicated. Similar and identical residues are highlighted in light and dark blue.

Figure S1

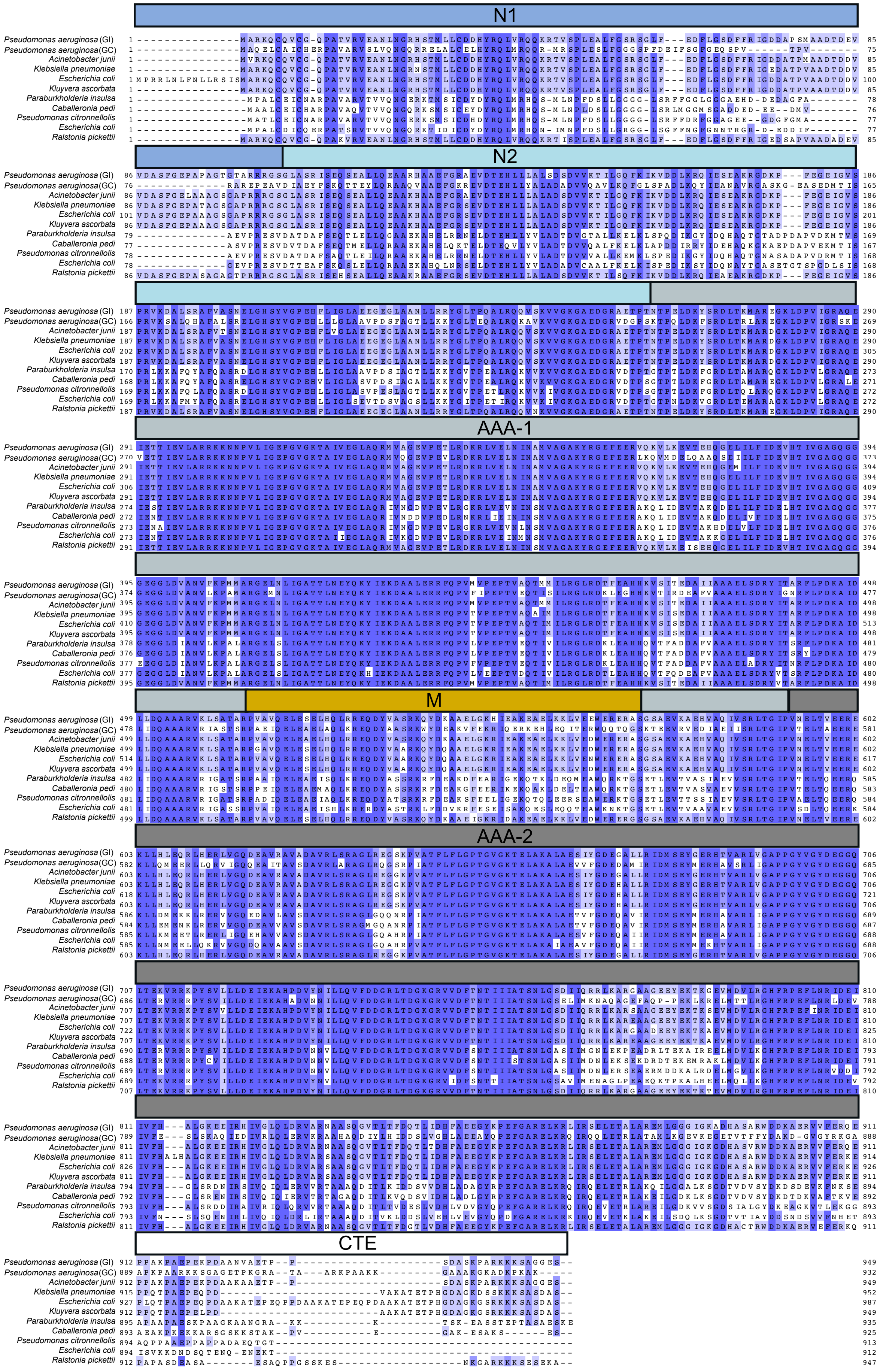

Figure S2

Sequence alignment of indicated ClpG homologs. The domain organization is indicated. Similar and identical residues are highlighted in light and dark blue.

Figure S2

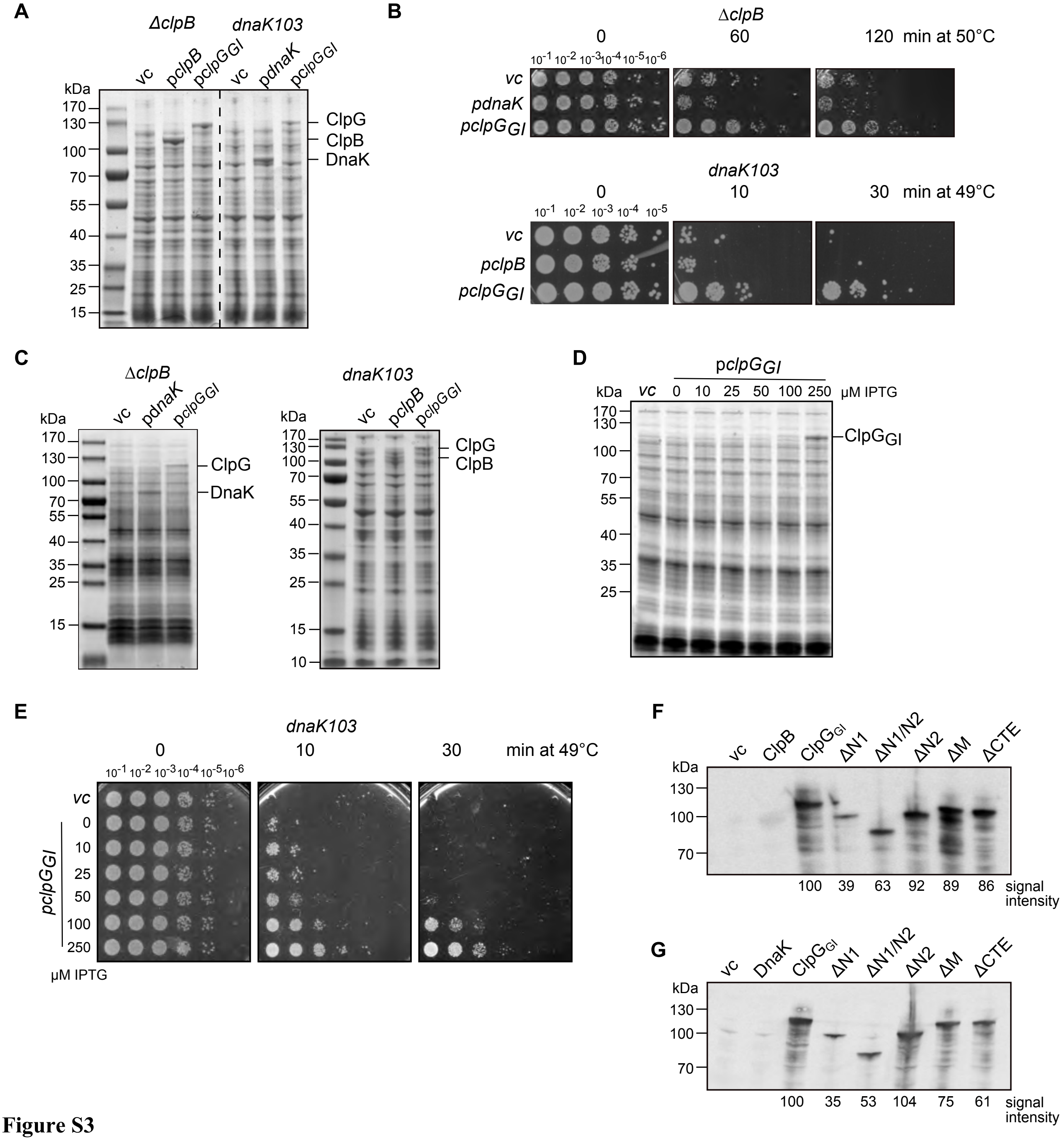

Figure S3

Chaperone production levels in heat resistance experiments. (A) Production levels of ClpB, DnaK and $ClpG_{GI}$ in *E. coli ΔclpB* and *dnaK103* cells upon addition of 100 µM IPTG were determined by SDS-PAGE. (B/C) Overexpression of DnaK or ClpB does not restore heat resistance of *E. coli ΔclpB* or *dnaK103* cells, respectively. *E. coli ΔclpB* or *dnaK103* cells harboring plasmids for expression of *E. coli dnaK, clpB* or *P. aeruginosa* $clpG_{GI}$ grown at 30°C to mid-logarithmic growth phase in presence of 100 µM IPTG and shifted to 50°C or 49°C. Serial dilutions (10_{-1} – $10^{-5/-6}$) of cells were prepared at the indicated time points, spotted on LB plates and incubated at 30°C for 24 h (B). Protein levels were determined by SDS-PAGE (C). (D/E) *E. coli dnaK103* mutant cells cells harboring plasmid-encoded *P. aeruginosa* $clpG_{GI}$ under control of an IPTG-inducible promoter were grown at 30°C in presence of the indicated IPTG concentrations. $ClpG_{GI}$ production levels were determined by SDS-PAGE (D). Cells were shifted to 49°C and serial dilutions (10_{-1} – 10^{--6}) of cells were prepared at the indicated time points, spotted on LB plates and incubated at 30°C for 24 h (E). vc: vector control. (F/G) Production levels of $ClpG_{GI}$ and deletion mutants in *E. coli ΔclpB* and *dnaK103* cells. *E. coli ΔclpB* (F) and *dnaK103* (G) cells harboring plasmids for expression of *E. coli clpB, dnaK* or *P. aeruginosa* $clpG_{GI}$ and its indicated deletion mutants (vc: empty vector control) were grown at 30°C for 1.5 h. Expression of $clpG_{GI}$ (wild type or mutants) was induced by addition of 100 µM IPTG for 2 h. Total cell extracts were prepared and levels of $GlpG_{GI}$ were determined by $GlpG_{GI}$ specific antibodies. Band intensities were quantified and set to 100 for $ClpG_{GI}$.

Figure S3

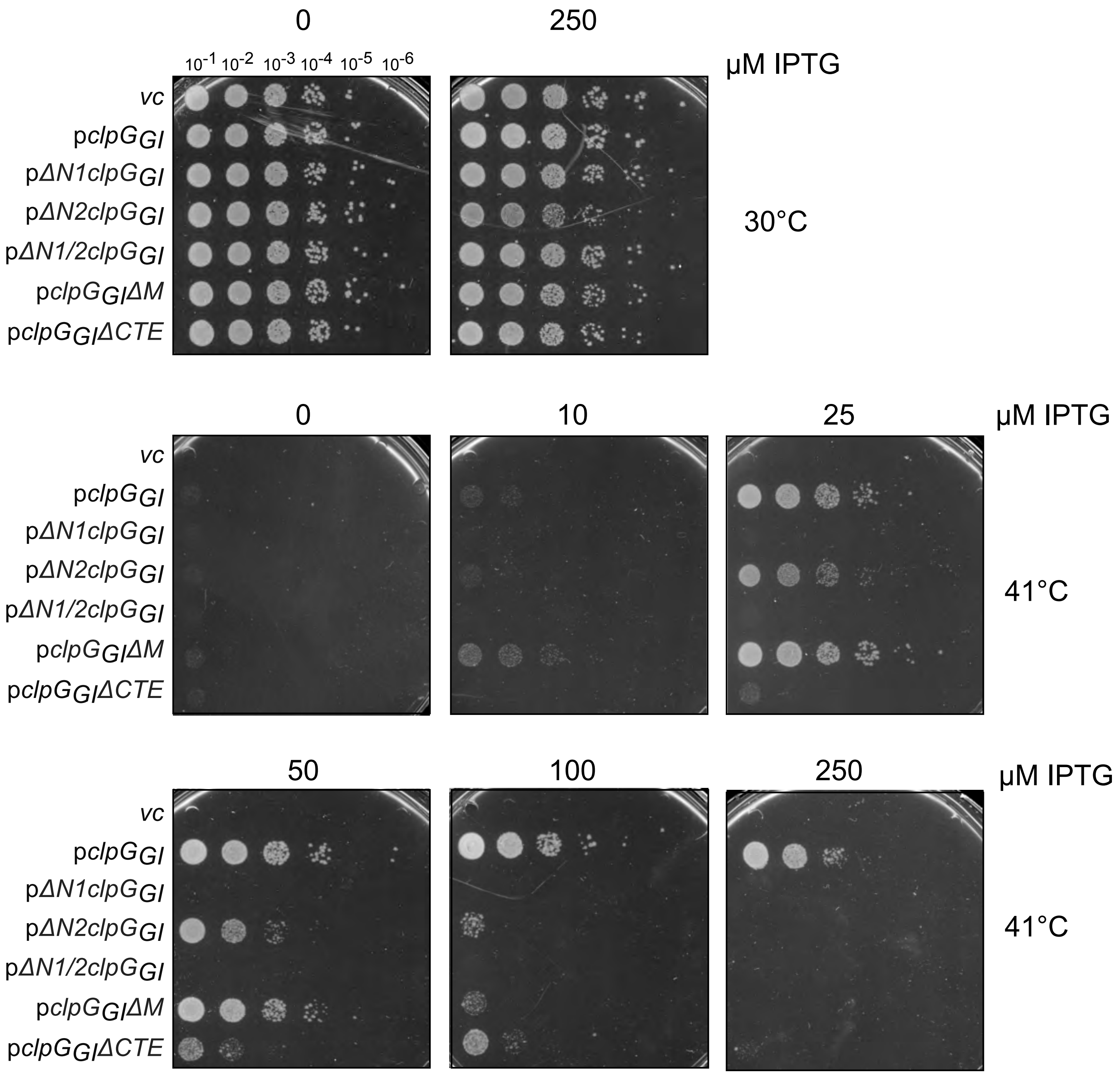

Figure S4

Serial dilutions (10^{-1} – 10^{-6}) of *E. coli dnaK103* mutant cells expressing *clpG$_{GI}$* or its indicated deletion variants were spotted on LB plates including the indicated IPTG concentrations. Plates were incubated at 30°C or 41°C for 24 h. vc: empty vector control

Figure S4

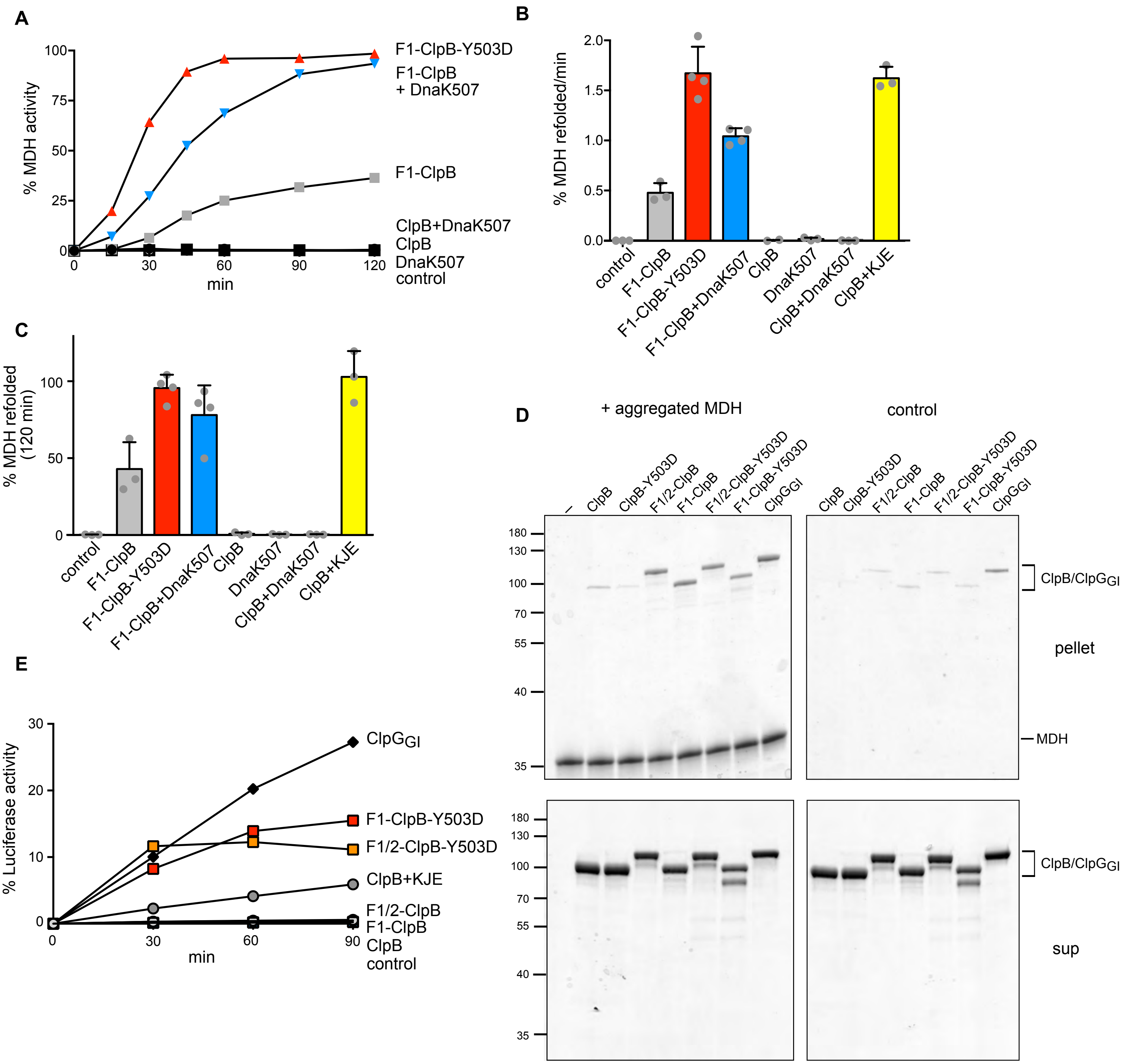

Figure S5

Fusion of N1-domain converts ClpB into a standalone disaggregase. (A) Disaggregation of aggregated Malate Dehydrogenase (MDH) by ClpB or the indicated ClpG$_{GI}$-ClpB chimeras in the absence or presence of DnaK507 was monitored by determining MDH activities at the indicated time point. The activity of native MDH was set at 100%. A control without chaperones is provided. **(B/C)** MDH refolding rates (B) and reactivation yields (120 min) (C) of indicated disaggregation reactions were determined were determined. KJE: DnaK chaperone system (DnaK/DnaJ/GrpE). Standard deviations are based on three independent experiments. (D) Heat aggregated MDH was incubated with ClpB, ClpG$_{GI}$ or the indicated ClpG$_{GI}$-ClpB chimeras in presence of 2 mM ATPγS. Soluble and insoluble (pellet) fractions were separated by centrifugation and analyzed by SDS-PAGE. A control experiment without MDH aggregates is provided. (E) Disaggregation of aggregated Luciferase by ClpG$_{GI}$, ClpB or the indicated ClpG$_{GI}$-ClpB chimeras (wt) was monitored by determining Luciferase activities at the indicated time point. The activity of Luciferase prior to heat denaturation was set at 100%. KJE: DnaK chaperone system.

Figure S5

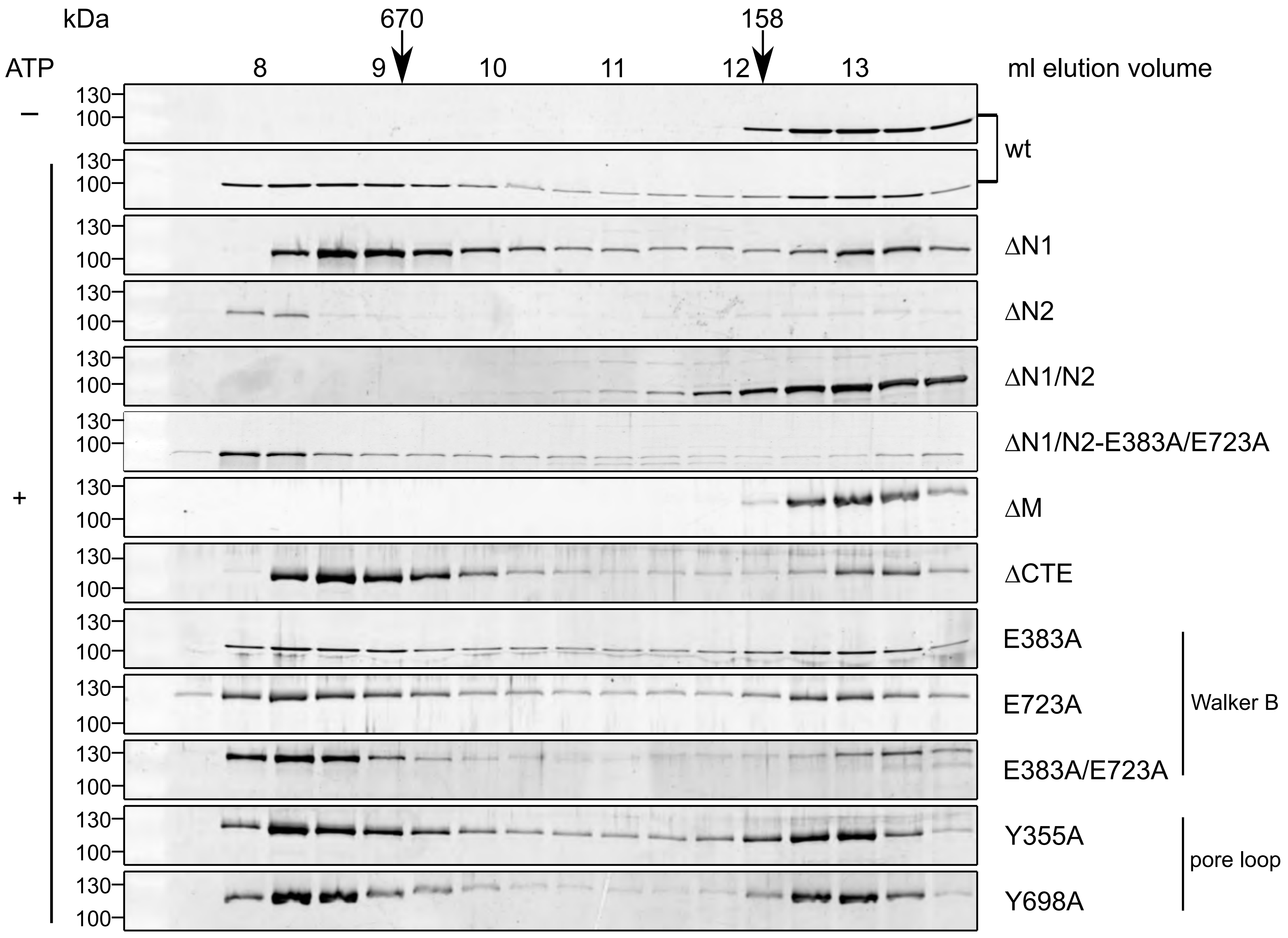

Figure S6

ATP-dependent oligomerization of $ClpG_{GI}$ wild type (wt) and indicated mutants. Oligomerization of $ClpG_{GI}$ was monitored by SEC runs in absence or presence of 2 mM ATP as indicated. Elution fractions were analyzed by SDS-PAGE. The elution positions of an SEC protein standard are indicated. Molecular weight markers for the SDS-PAGEs are provided.

Figure S6

A

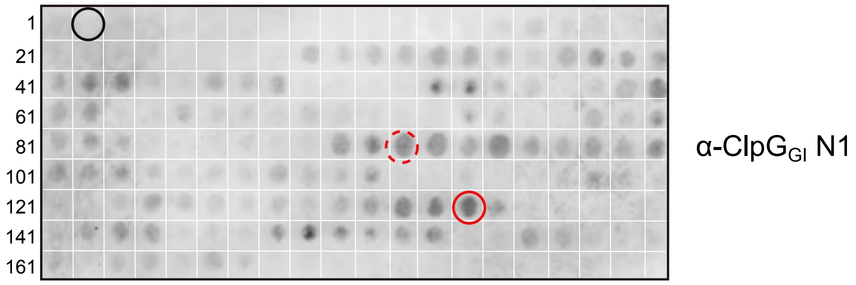

B

1 ATPSNKRSRDSES
2 SNKRSRDSESTEE
3 RSRDSESTEEPVV
4 DSESTEEPVVDEK
5 STEEPVVDEKSTS
6 EPVVDEKSTSKQN
7 VDEKSTSKQNNAA
8 KSTSKQNNAAPEG
9 SKQNNAAPEGEQT
10 NNAAPEGEQTTCV
11 APEGEQTTCVEKF
12 GEQTTCVEKFEEL
13 TTCVEKFEELKLS
14 VEKFEELKLSQPT
15 FEELKLSQPTLKA
16 LKLSQPTLKAIEK
17 SQPTLKAIEKMGF
18 TLKAIEKMGFTTM
19 AIEKMGFTTMTSV
20 KMGFTTMTSVQAR
21 FTTMTSVQARTIP
22 MTSVQARTIPPLL
23 VQARTIPPLLAGR
24 RTIPPLLAGRDVL
25 PPLLAGRDVLGAA
26 LAGRDVLGAAKTG
27 RDVLGAAKTGSGK
28 LGAAKTGSGKTLA
29 AKTGSGKTLAFLI
30 GSGKTLAFLIPAI
31 KTLAFLIPAIELL
32 AFLIPAIELLHSL
33 IPAIELLHSLKFK
34 IELLHSLKFKPRN
35 LHSLKFKPRNGTG
36 LKFKPRNGTGIIV
37 KPRNGTGIIVITP
38 NGTGIIVITPTRE
39 GIIVITPTRELAL
40 VITPTRELALQIF
41 PTRELALQIFGVA
42 ELALQIFGVAREL
43 LQIFGVARELMEF
44 FGVARELMEFHSQ
45 ARELMEFHSQTFG
46 LMEFHSQTFGIVI
47 FHSQTFGIVIGGA
48 QTFGIVIGGANRR
49 GIVIGGANRRQEA
50 IGGANRRQEAEKL
51 ANRRQEAEKLMKG
52 RQEAEKLMKGVNM
53 AEKLMKGVNMLIA
54 LMKGVNMLIATPG
55 GVNMLIATPGRLL
56 MLIATPGRLLDHL
57 ATPGRLLDHLQNT
58 GRLLDHLQNTKGF
59 LDHLQNTKGFVFK
60 LQNTKGFVFKNLK
61 TKGFVFKNLKALI
62 FVFKNLKALIIDE
63 KNLKALIIDEADR
64 KALIIDEADRILE
65 IIDEADRILEIGF
66 EADRILEIGFEDE
67 RILEIGFEDEMRQ
68 EIGFEDEMRQIIK
69 FEDEMRQIIKILP
70 EMRQIIKILPNED
71 QIIKILPNEDRQS
72 KILPNEDRQSMLF
73 PNEDRQSMLFSAT
74 DRQSMLFSATQTT
75 SMLFSATQTTKVE
76 FSATQTTKVEDLA
77 TQTTKVEDLARIS
78 TKVEDLARISLRP
79 EDLARISLRPGPL
80 ARISLRPGPLFIN
81 SLRPGPLFINVVP
82 PGPLFINVVPETD
83 LFINVVPETDNST
84 NVVPETDNSTADG
85 PETDNSTADGLEQ
86 DNSTADGLEQGYV
87 TADGLEQGYVVCD
88 GLEQGYVVCDSDK
89 QGYVVCDSDKRFL
90 VVCDSDKRFLLLF
91 DSDKRFLLLFSFL
92 KRFLLLFSFLKRN
93 LLLFSFLKRNQKK
94 FSFLKRNQKKKII
95 LKRNQKKKIIVFL
96 NQKKKIIVFLSSC
97 KKIIVFLSSCNSV
98 IVFLSSCNSVKYY
99 LSSCNSVKYYAEL
100 CNSVKYYAELLNY
101 VKYYAELLNYIDL
102 YAELLNYIDLPVL
103 LLNYIDLPVLELH
104 YIDLPVLELHGKQ
105 LPVLELHGKQKQQ
106 LELHGKQKQQKRT
107 HGKQKQQKRTNTF
108 QKQQKRTNTFFEF
109 QKRTNTFFEFCNA
110 TNTFFEFCNAERG
111 FFEFCNAERGILI
112 FCNAERGILICTD
113 AERGILICTDVAA
114 GILICTDVAARGL
115 ICTDVAARGLDIP
116 DVAARGLDIPAVD
117 ARGLDIPAVDWII
118 LDIPAVDWIIQFD
119 PAVDWIIQFDPPD
120 DWIIQFDPPDDPR
121 IQFDPPDDPRDYI
122 DPPDDPRDYIHRV
123 DDPRDYIHRVGRT
124 RDYIHRVGRTARG
125 IHRVGRTARGTKG
126 VGRTARGTKGKGK
127 TARGTKGKGKSLM
128 GTKGKGKSLMFLT
129 GKGKSLMFLTPNE
130 KSLMFLTPNELGF
131 MFLTPNELGFLRY
132 TPNELGFLRYLKA
133 ELGFLRYLKASKV
134 FLRYLKASKVPLN
135 YLKASKVPLNEYE
136 ASKVPLNEYEFPE
137 VPLNEYEFPENKI
138 NEYEFPENKIANV
139 EFPENKIANVQSQ
140 ENKIANVQSQLEK
141 IANVQSQLEKLIK
142 VQSQLEKLIKSNY
143 QLEKLIKSNYYLH
144 KLIKSNYYLHQTA
145 KSNYYLHQTAKDG
146 YYLHQTAKDGYRS
147 HQTAKDGYRSYLQ
148 AKDGYRSYLQAYA
149 GYRSYLQAYASHS
150 SYLQAYASHSLKT
151 QAYASHSLKTVYQ
152 ASHSLKTVYQIDK
153 SLKTVYQIDKLDL
154 TVYQIDKLDLAKV
155 QIDKLDLAKVAKS
156 KLDLAKVAKSYGF
157 LAKVAKSYGFPVP
158 VAKSYGFPVPPKV
159 SYGFPVPPKVNIT
160 FPVPPKVNITIGA
161 PPKVNITIGASGK
162 VNITIGASGKTPN
163 TIGASGKTPNTKR
164 ASGKTPNTKRRKT
165 GKTPNTKRRKTHK
166 AKTLILSHLRFVV
167 VVHIARNYAGYG

C

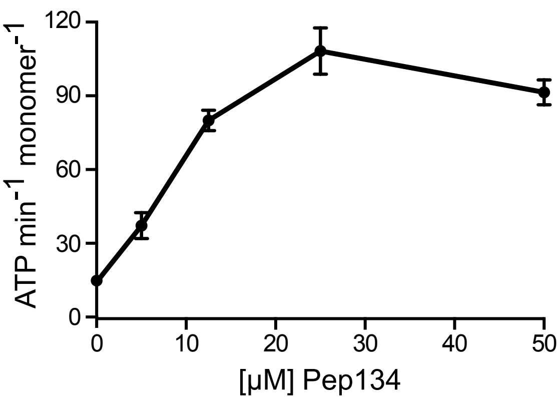

D

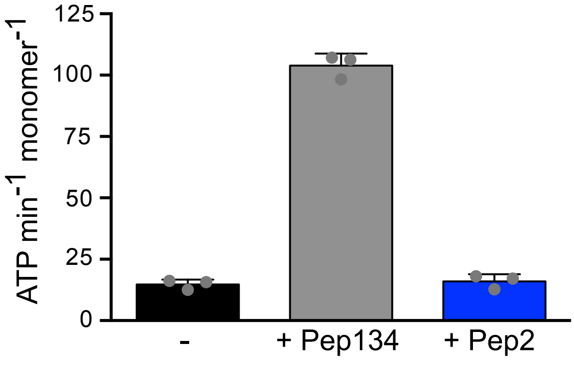

Figure S7

The ClpG$_{GI}$ interacting peptide Pep134 stimulates ClpG$_{GI}$ ATPase activity. (A) Binding of the ClpG$_{GI}$ N1 domain to a Has1 scanning peptide library. ClpG$_{GI}$ N1 domain was incubated with the peptide library and bound N1 domain was detected by immunoblot analysis after electrotransfer to a PVDF membrane using N1-specific antibodies. Pep134 is highlighted by a red circle. The alternative interaction peptide Pep92 is indicated by a dashed red circle. Peptide numbers are indicated. (B) Sequences of 13-mer peptides overlapping by 10 residues covering the sequence of *S. cerevisiae* Has1. Sequences of Pep92 and Pep134 are highlighted. (C) ATPase activity of ClpG$_{GI}$ was determined in presence of increasing concentrations of Pep134.

Figure S7

Table S1: strains and plasmids used in this study

Strain	Description	Source or reference
E. coli XL1 blue	*recA1 endA1 gyrA96 thi-1 hsdR1 supE44 relA1 lac* [F' *proAB lacI^q ΔM15* Tn10 (Tcr)]	Stratagene
E. coli BL21	*F- ompT lon hsdSB gal dcm λ* (DE3)	Novagen
E. coli ΔclpB	MC4100 Δ*clpB*::Km	(1)
E. coli dnaK103	MC4100 *dnaK103 (dnaK ts mutant)*	(2)
**Plasmid**	**Description**	**Source or reference**
pUHE21	Vector for IPTG-inducible gene expression in *E.coli*	(3)
pUHE21-*clpG$_{GI}$*	Vector for IPTG-inducible expression of clpG$_{GI}$ in *E.coli* Δ*clpB* and *dnaK103* cells	(2)
pUHE21-*clpB*	Vector for IPTG-inducible expression of *clpB* in *E.coli* Δ*clpB* cells	(1)
pUHE21-*dnaK*	Vector for IPTG-inducible expression of *dnaK* in *E.coli* *dnaK103* cells	(3)
pUHE21-Δ*N1-clpG$_{GI}$*	Vector for IPTG-inducible expression of Δ*N1-clpG$_{GI}$* (Δ1-106) in *E.coli* Δ*clpB* and *dnaK103* cloned into *BamHI* and *XbaI* sites	This study
pUHE21-Δ*N2-clpG$_{GI}$*	Vector for IPTG-inducible expression of Δ*N2-clpG$_{GI}$* (Δ108-247) in *E.coli* Δ*clpB* and *dnaK103* cells	This study
pUHE21-Δ*N1/N2-clpG$_{GI}$*	Vector for IPTG-inducible expression of Δ*N1/N2-clpG$_{GI}$* (Δ1-255) in *E.coli* Δ*clpB* and *dnaK103* cells	This study
pUHE21-Δ*M-clpG$_{GI}$*	Vector for IPTG-inducible expression of Δ*M-clpG$_{GI}$* (Δ516-562 plus Gly-Gly linker) in *E.coli* Δ*clpB* and *dnaK103* cells	This study
pUHE21-Δ*CTE-clpG$_{GI}$*	Vector for IPTG-inducible expression of Δ*CTE-clpG$_{GI}$* (Δ912-949) in *E.coli* Δ*clpB* and *dnaK103* cells	This study
pET24a-*clpG$_{GI}$*	Vector for IPTG-inducible expression of *clpG$_{GI}$* in *E.coli* BL21 cells	(2)
pET24a-Δ*N1-clpG$_{GI}$*	Vector for IPTG-inducible expression of Δ*N1-clpG$_{GI}$* (Δ1-106) in *E.coli* BL21 cells	(2)
pET24a-Δ*N2-clpG$_{GI}$*	Vector for IPTG-inducible expression of Δ*N2-clpG$_{GI}$* (Δ108-247) in *E.coli* BL21 cells	This study
pET24a-Δ*N1/N2-clpG$_{GI}$*	Vector for IPTG-inducible expression of Δ*N1/N2-clpG$_{GI}$* (Δ1-247) in *E.coli* BL21 cells	This study
pET24a-Δ*M-clpG$_{GI}$*	Vector for IPTG-inducible expression of Δ*M-clpG$_{GI}$* (Δ516-562 plus Gly-Gly linker) in *E.coli* BL21 cells	This study
pET24a-Δ*CTE-clpG$_{GI}$*	Vector for IPTG-inducible expression of *-clpG$_{GI}$-ΔCTE* (Δ912-949) in *E.coli* BL21 cells	This study
pET24a-*clpG$_{GI}$*-E383A	Vector for IPTG-inducible expression of pET24a-*clpG$_{GI}$*-E383A in *E.coli* BL21 cells	This study
pET24a-*clpG$_{GI}$*-E723A	Vector for IPTG-inducible expression of pET24a-*clpG$_{GI}$*-E723A in *E.coli* BL21 cloned into *NdeI* and *XhoI* sites	This study
pET24a-*clpG$_{GI}$*-E383A/E723A	Vector for IPTG-inducible expression of pET24a-*clpG$_{GI}$*-E383A/E723A in *E.coli* cells	This study
pET24a-*clpG$_{GI}$*-Y355A	Vector for IPTG-inducible expression of pET24a-*clpG$_{GI}$*-Y355A in *E.coli* BL21 cells	This study
pET24a-*clpG$_{GI}$*-Y698A	Vector for IPTG-inducible expression of pET24a-*clpG$_{GI}$*-Y698A in *E.coli* BL21 cells	This study
pET24a-N1-domain-*clpG$_{GI}$*	Vector for IPTG-inducible expression of pET24a-*clpG$_{GI}$* (1-82) harboring a C-terminal Twin-Strep-Tag in *E. coli* BL21 cells	This study
pET24a-*F1-clpB*	Vector for IPTG-inducible expression of *F1-clpB (clpB*	This study

	(Δ1-145) fused with the N1-domain of *clpG*$_{GI}$ (1-107)) in *E.coli* BL21 cells	
pET24a-*F2-clpB*	Vector for IPTG-inducible expression of *F1/2-clpB* (Δ*N-clpB* (Δ1-145) fused with the N1/N2-domains of *clpG*$_{GI}$ (1-248)) in *E.coli* BL21 cells	This study
pET24a-*F1-clpB*-Y503D	Vector for IPTG-inducible expression of *F1-clpB*-Y503D (Δ*N-clpB*-Y503D (Δ1-145) fused with the N1-domain of *clpG*$_{GI}$ (1-107)) in *E.coli* BL21 cells	This study
pET24a-F1/*2-clpB*-Y503D	Vector for IPTG-inducible expression of F1/2-clpB-Y503D (Δ*N-clpB*-Y503D (Δ1-145) fused with the N1/N2-domain of *clpG*$_{GI}$ (1-247)) in *E.coli* BL21 cells	This study
pET24a-*dnaK507*	Vector for IPTG-inducible expression of *dnaK507* (Δ508-638) in *E.coli* BL21 cells	This study
pDS56-*clpB*	Vector for IPTG-inducible expression of *clpB-C*$_{His6}$ in *E.coli* Δ*clpB* cells	(4)
pDS56-*clpB*-Y503D	Vector for IPTG-inducible expression of *clpB-Y503-C*$_{His6}$ in *E.coli* Δ*clpB* cells	(4)

References

1. Mogk, A., Schlieker, C., Strub, C., Rist, W., Weibezahn, J., and Bukau, B. (2003) Roles of individual domains and conserved motifs of the AAA+ chaperone ClpB in oligomerization, ATP-hydrolysis and chaperone activity. *J Biol Chem* **278**, 15-24
2. Lee, C., Franke, K. B., Kamal, S. M., Kim, H., Lunsdorf, H., Jager, J., Nimtz, M., Trcek, J., Jansch, L., Bukau, B., Mogk, A., and Romling, U. (2018) Stand-alone ClpG disaggregase confers superior heat tolerance to bacteria. *Proc Natl Acad Sci U S A* **115**, E273-E282
3. Mayer, M. P., Schroder, H., Rudiger, S., Paal, K., Laufen, T., and Bukau, B. (2000) Multistep mechanism of substrate binding determines chaperone activity of Hsp70. *Nat Struct Biol* **7**, 586-593
4. Oguchi, Y., Kummer, E., Seyffer, F., Berynskyy, M., Anstett, B., Zahn, R., Wade, R. C., Mogk, A., and Bukau, B. (2012) A tightly regulated molecular toggle controls AAA+ disaggregase. *Nat Struct Mol Biol* **19**, 1338-1346
